# Thigh phlegmon as a first sign of a ruptured pelvic presacral abscess caused by ileal diverticulum fistula: A case report

**DOI:** 10.1016/j.ijscr.2021.105836

**Published:** 2021-03-26

**Authors:** Dainius Simcikas, Alisa Maksimova-Cesnaviciene, Mindaugas Gvazdaitis, Jonas Jurgaitis, Agne Cizauskaite, Narimantas Evaldas Samalavicius

**Affiliations:** a Klaipeda University Hospital, 41 Liepojos Str., LT-92288, Klaipeda, Lithuania; b Department of Nursing, Faculty of Health Sciences, Klaipeda University, 84 Herkaus Manto Str., LT-92294, Klaipeda, Lithuania; c Klaipeda Republican Hospital, Day Surgery Center, 9 Puodziu Str., LT-92127, Klaipeda, Lithuania; d Lithuanian University of Health Sciences, 9 A. Mickeviciaus Str., LT-44307, Kaunas, Lithuania; e Institute of Clinical Medicine, Medical Faculty, Vilnius University, 2 Santariskiu Str., LT-08410, Vilnius, Lithuania; f Health Research and Innovation Science Center, Faculty of Health Sciences, Klaipeda University, 84 Herkaus Manto Str., LT-92294, Klaipeda, Lithuania

**Keywords:** Ileal diverticulum, Presacral abscess, Thigh phlegmon, Extra-pelvic pathways, Case report

## Abstract

**Introduction:**

Ileal diverticula usually remain asymptomatic. If complicated, they may present as intra-abdominal or pelvic abscess. Abscess formation in the presacral space is very rare. The rupture of abscess and spread of pus to extra-pelvic sites through anatomical structures of the pelvis is extremely rare. It carries high mortality if not diagnosed on time.

**Case presentation:**

We report a case of 52-year-old woman presented with thigh phlegmon and septic condition. The CT scan revealed free air in the left leg and pelvic presacral fluid collection descending to extra-pelvic direction through the greater sciatic notch. Moreover, ileal fistula to presacral abscess was suspected. Multiple incisions and fasciotomies were urgently performed to treat thigh phlegmon. Subsequently, laparotomy was carried out and ileal fistula was excised. Histological examination of the surgical specimen demonstrated that the fistula to presacral abscess has formed due to perforated ileal diverticulum. 3 years after the surgery the patient remains healthy without recurrence.

**Discussion:**

Rupture of presacral abscess to extra-pelvic site due to ileal diverticulum fistula is an extremely rare case, to our best knowledge, never reported in literature. Due to a rare occurrence and early septic complications if diagnosed late, this condition carries a high mortality rate.

**Conclusion:**

Intrapelvic pathology must be considered in patients with thigh phlegmon in order to prevent complications and associated mortality.

## Highlights

• Ileal diverticulum rarely causes pelvic abscess.
• Pelvic abscess rarely causes thigh phlegmon.
• Rupture of presacral abscess to extra-pelvic site carries high mortality.
• Intrapelvic pathology has to be considered in patients with thigh phlegmon.

## 1 Introduction

Small intestine diverticula are rare, accounting only for up to 1.3% of all intestinal diverticulosis cases [1]. Diverticula may be acquired or congenital [2]. Meckel’s diverticulum is the most common congenital abnormality of gastrointestinal tract [3]. While in most cases presence of diverticula remains asymptomatic, up to 30% of patients tend to develop complications like perforation, abscess formation, bowel obstruction or formation of a fistula [1,2,4,5].

Pelvic abscess in peritoneal cavity is quite common complication of abdominal pathology [6]. However, presacral abscesses are uncommon and may present with vague and diverse clinical features. In most cases the only sign of the presacral abscess is a lumbar or abdominal pain accompanied by fever [7].

Though symptoms of the complicated diverticula and presacral abscess are variable and nonspecific, the extra-pelvic manifestation is very rare. It may follow several routes, including greater or lesser sciatic notch, obturator foramen, inguinal or femoral canal, presenting as an extra-pelvic abscess or phlegmon [8,9]. Miscellaneous presentation of ileal diverticula and presacral abscesses frequently leads to delayed diagnosis or misdiagnosis, resulting in higher incidence of mortality [2,4,5].

In this report, we present an extremely rare case of presacral abscess manifestation as thigh phlegmon due to the ileal diverticulum fistula. To our best knowledge, there are no reported cases of ileal diverticulum fistula presenting in such a fashion. This case is reported in consensus with SCARE 2020 guidelines [10].

## 2 Case presentation

A 52-year-old woman presented to the emergency room with intense pain and swelling of the left leg and lumbar area, accompanied by high fever. According to the patient, the pain occurred 6 days ago without traumatic events. After assessing the patient’s medical history it was known that 23 years ago she delivered a baby via Caesarean section. Unfortunately, an early postoperative period was complicated: laparotomy, hysterectomy and cholecystectomy were performed due to pelvic abscess formation and peritonitis. At a subsequent time the patient remained healthy until her current hospitalization.

Clinical evaluation revealed swelling of the previously mentioned areas and painful palpation. There was significant redness in the lateral middle part of the left thigh. Initial blood workup showed elevated levels of serum C-reactive protein (518 mg/L) and procalcitonin (4.4 ng/mL). Due to a septic condition, the patient was admitted to the intensive care unit.

**Fig. 1:**
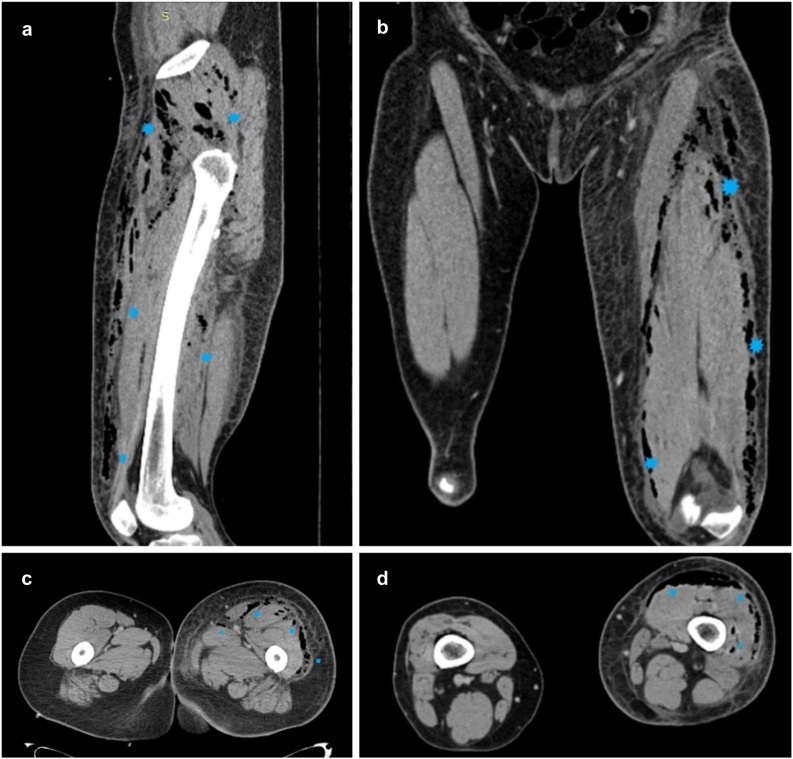
Sagittal (**a**), coronal (**b**) and axial (**c, d**) CT images of the left thigh. *Blue stars* mark multiple gas density areas (phlegmon) distributed mainly in soft tissues of anterior thigh compartment and gluteal region.

**Fig. 2:**
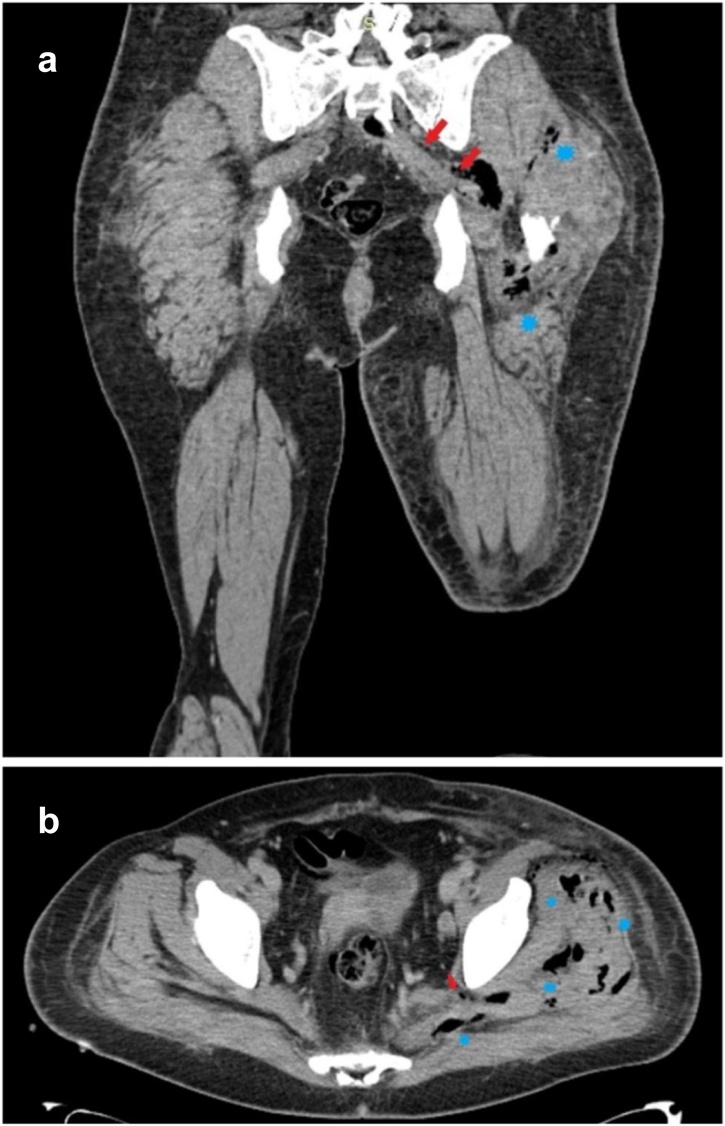
Coronal (**a**) and axial (**b**) CT images of inferior part of pelvis and gluteal region. *Blue stars* mark free gas in soft tissues and muscles around left hip area (phlegmon). *Red arrows* show continuity of gas interpositions from pelvis to soft tissues through the greater sciatic notch.

**Fig. 3:**
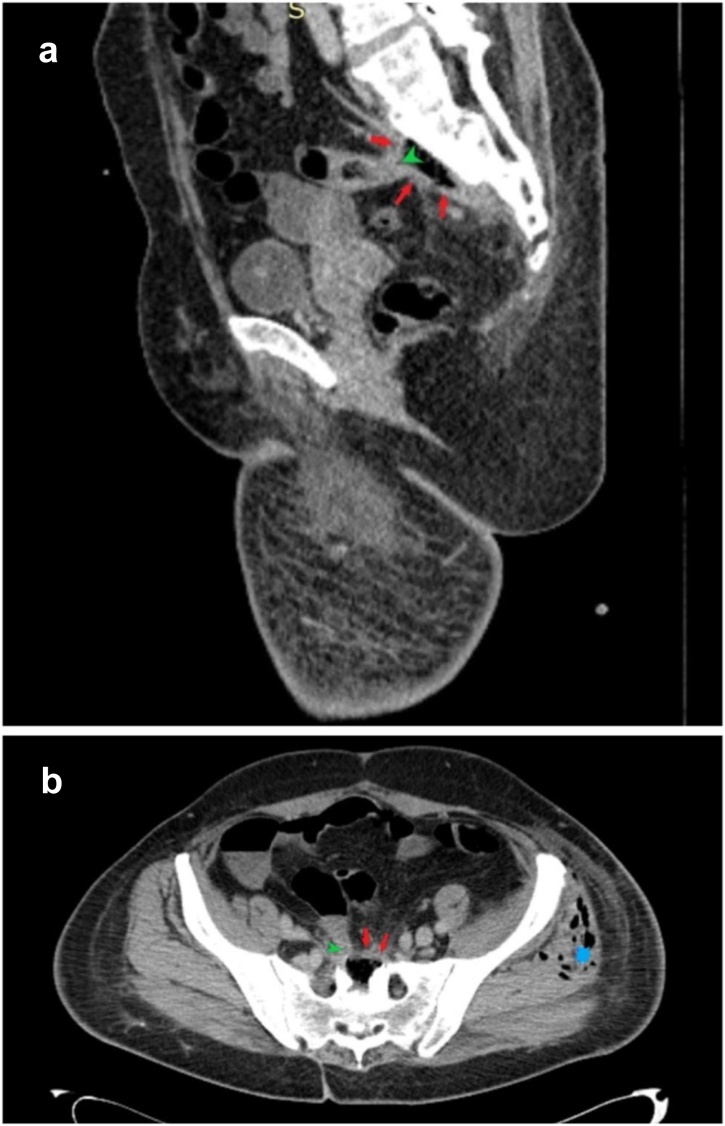
Sagittal (**a**) and axial (**b**) CT images of pelvis and presacral region. *Blue star* shows intramuscular gas regions (phlegmon). *Red arrows* mark presacral collection of gas interpreted as presacral abscess. *Green arrowheads* show ileal segment fixated to the anterior wall of presacral abscess, interpreted as fistula site.

For further evaluation, a computed tomography (CT) scan was performed. It revealed free air in the left leg (Fig. 1) and presacral abscess draining to the left gluteal area and thigh through the greater sciatic notch (Fig. 2). Moreover, it showed fixated ileum to the abscess. To treat thigh phlegmon, multiple incisions and fasciotomies were performed in the left gluteus and thigh, releasing a large amount of purulent discharge. Thigh phlegmon gradually resolved, however, the origin of the presacral abscess was still unknown. CT data (Fig. 3) and postsurgical colonoscopy with terminal ileoscopy raised suspicion of an ileal fistula causing bowel content evacuation to the presacral space. The second surgery was performed. Ileal diverticulum was found approximately 10 cm from ileocecal valve. A fistula was connecting the lumen of diverticulum with presacral abscess. Ileal fistulectomy and presacral abscessotomy were performed. Postoperative course was uneventful and the patient was discharged from the hospital. Histological examination of the surgical specimen identified the perforated ileal diverticulum, forming fistula to the presacral abscess. According to the pathology report, the ileal diverticulum was more likely congenital.

## 3 Discussion

A thorough search of the databases did not reveal any similar reports about small bowel diverticula, accompanied by pelvic abscess and presenting with extra-abdominal symptoms. A handful of case reports were presenting a complicated large bowel diverticulitis occurring as necrotizing fasciitis in extra-abdominal sites.

Ileal diverticula are mostly found incidentally during radiological investigations, laparotomy or autopsy [1,4]. In most cases, patients remain asymptomatic or present with nonspecific symptoms like chronic abdominal pain, vomiting, alternating diarrhea, constipation. In our case, the patient did not report any chronic symptoms [1,2,4,5].

Presacral abscesses are uncommon, mostly originate after rectal surgery [7,11]. In our case there was a newly formed presacral abscess. However, the erosion of sacral bone observed in CT pictures, suggested the abscess cavity formed a long time ago. It is very debatable, whether it could be the consequence of a previously formed abscess after performed hysterectomy 23 years ago. We found no reports concerning presacral abscess formation following gynecological surgery.

Although CT scan showed fixated ileum to the abscess and the colonoscopy with terminal ileoscopy raised suspicion of ileal fistula, the diagnosis was doubtful since none similar cases have been reported before. It became clear after the second surgery when the fistulated ileal diverticulum was found, excised and verified histologically. Perhaps chronic inflammation in the old pelvic presacral cavity led to fistula formation of the ileal diverticulum, resulting in new abscess formation. Pathology report suggested the origin of the diverticulum to be more likely congenital. According to literature, the most common congenital anomaly is Meckel’s diverticulum. Usually it is located at a distal ileum approximately 60 cm from the ileocecal valve [3]. In our case, we did not classify the diverticulum as Meckel’s as it was found 10 cm from ileocecal valve.

Spread of intra-abdominal infection to extra-abdominal sites carries a high mortality rate if diagnosed late [9]. Fortunately, we managed to stop the further spread of the infection, thus preventing unwanted or lethal events. 3 years after the surgery the patient remains healthy without any signs of recurrent disease.

## 4 Conclusion

The purpose of presenting this case is to raise awareness of extremely rare manifestations of complicated small intestine diverticula and pelvic abscesses. Although rupture of the presacral abscess to the thigh due to ileal diverticulum fistula is extremely rare, intrapelvic pathology has to be considered in patients with thigh phlegmon. An early diagnosis and appropriate interventions are crucial for reducing the incidence of further complications and the associated mortality.

## Declaration of Competing Interest

The authors declare that they have no conflicts of interest.

## Sources of funding

Not applicable.

## Ethical approval

Authors were complying with Klaipeda university hospital requirements. The data used are anonymous and patient rights are preserved.

## Consent

Each patient signs general form of informed consent while admitted to Klaipeda university hospital, and item number 5 states the following: “I am informed that Klaipeda university hospital is scientific and teaching institution, and I agree to participate in teaching process during my treatment here, and I agree that anonymous data concerning my health can be used for teaching and scientific purposes.” Therefore, this form enables us to collect and use the data.

Written informed consent was obtained from the patient for publication of this case report and accompanying images. A copy of the written consent is available for review by the Editor-in-Chief of this journal on request.

## Author contribution

All authors contributed to the article conception and design. Material preparation, data collection and analysis were performed by Dainius Simcikas, Alisa Maksimova-Cesnaviciene and Mindaugas Gvazdaitis. The first draft of the manuscript was written by Dainius Simcikas and all authors commented on previous versions of the manuscript. All authors read and approved the final manuscript.

## Registration of research studies

Not applicable.

## Guarantor

Dainius Simcikas.

## Provenance and peer review

Not commissioned, externally peer-reviewed.

## Availability of data and material

No data available.

## Code availability

Not applicable.
